# OPCs eat in response to sensory experiences

**DOI:** 10.1038/s42003-022-04099-w

**Published:** 2022-10-19

**Authors:** Karli Montague-Cardoso

**Affiliations:** Communications Biology, https://www.nature.com/commsbio/

## Abstract

Oligodendrocytes are derived from a subtype of glia called oligodendrocyte precursor cells (OPCs). The potential functions of OPCs beyond oligodendrogenesis however, have remained elusive. In their latest study, Auguste et al. demonstrate that OPCs could play a regulatory role in synaptic connectivity in the developing and adult mouse visual cortex - a function that is independent of oligodendrogenesis.

Oligodendrocyte precursor cells (OPCs) are a subtype of glial cell that differentiate into oligodendrocytes. Other than being precursors for oligodendrocytes however, the question of whether OPCs possess additional, independent functions has so far remained largely unanswered. A recent study from Auguste et al.^1^, begins to shed some light on this question. They use confocal microscopy and fluorescence in situ hybridization to elegantly visualise OPCs in the developing and adult mouse visual cortex. By doing so, they show that OPCs are likely to play a key role in regulating experience-dependent synaptic refinement. Specifically, they reveal that OPCs are capable of engulfing presynaptic thalamocortical inputs to the primary visual cortex during critical developmental windows in which refinement is heightened. Through a series of imaging studies, they present evidence that the engulfed synapses are likely to be degraded within phagosomal compartments. Auguste et al. then go on to show that not only does this engulfment appear to be heightened by sensory experience (using a visual deprivation/stimulation paradigm), but is also affected by the depletion of microglia, which are already known to play a crucial role in synaptic phagocytosis. This strongly suggests the existence of microglial-OPC communication in the context of synaptic refinement.

Overall, this study makes an intriguing contribution to our understanding of how OPCs respond to sensory experiences. Whilst future studies will need to address remaining unknowns such as whether sensory experience changes the rate at which OPC engulf synapses, the finding that OPCs can influence synapse number provides valuable insights into developmental and disease mechanisms in the brain.

**Figure:**
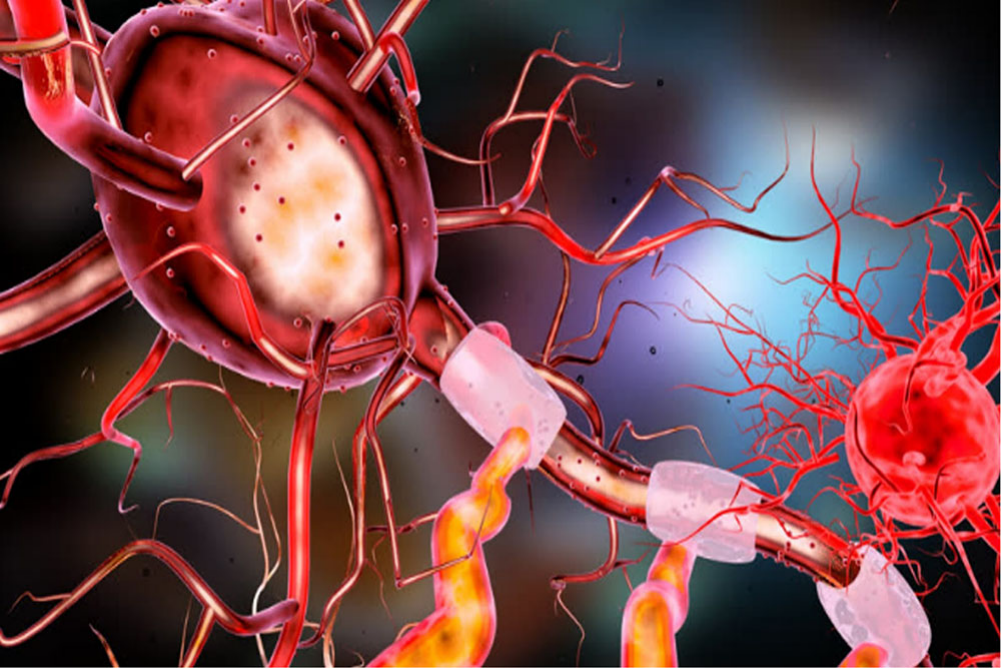
Pixabay
